# Remediation of Cr(VI)-Contaminated Soil by Biochar-Supported Nanoscale Zero-Valent Iron and the Consequences for Indigenous Microbial Communities

**DOI:** 10.3390/nano12193541

**Published:** 2022-10-10

**Authors:** Jianwei Yang, Xiangpeng Tan, Muhammad Shaaban, Yajun Cai, Buyun Wang, Qi’an Peng

**Affiliations:** 1 School of Environmental Engineering, Wuhan Textile University, Wuhan 430073, China; 2 Key Laboratory of Mountain Surface Processes and Ecological Regulation, Institute of Mountain Hazards and Environment, Chinese Academy of Sciences, Chengdu 610041, China

**Keywords:** soil remediation, hexavalent chromium, biochar, nanoscale zero-valent iron, soil microecology

## Abstract

Biochar/nano-zero-valent iron (BC-nZVI) composites are currently of great interest as an efficient remediation material for contaminated soil, but their potential to remediate Cr-contaminated soils and effect on soil microecology is unclear. The purpose of this study was to investigate the effect of BC-nZVI composites on the removal of Cr(VI) from soil, and indigenous microbial diversity and community composition. The results showed that after 15 days of remediation with 10 g/kg of BC-nZVI, 86.55% of Cr(VI) was removed from the soil. The remediation of the Cr-contaminated soil with BC-nZVI resulted in a significant increase in OTUs and α-diversity index, and even a significant increase in the abundance and diversity of indigenous bacteria and unique bacterial species in the community by reducing the toxic concentration of Cr, changing soil properties, and providing habitat for survival. These results confirm that BC-nZVI is effective in removing Cr(VI) and stabilizing Cr in soil with no significant adverse effects on soil quality or soil microorganisms.

## 1. Introduction

A large number of chromium-containing wastes are currently discharged into water bodies and soil, causing serious pollution [1,2]. Chromium (Cr) pollutants are highly toxic, carcinogenic, and can directly or indirectly endanger people’s health through the food chain [3,4]. Therefore, the harmless treatment of Cr and its pollution management have attracted widespread attention [5].

Conversion of Cr(VI) to Cr(III) and formation of stable compounds in soils are widely used remediation methods with the advantages of high treatment efficiency and low cost [6]. Iron-based composites, which are highly reducible and environmentally friendly, have been widely used for the remediation of Cr(VI)-contaminated soils. Singh et al. [7] documented that the addition of 0.10 g/L nZVI to a Cr-contaminated soil from a tannery in Kanpur, India, resulted in complete reduction in Cr(VI) within 120 min. However, the application of nZVI is largely limited by its tendency to agglomerate and passivate, and its tendency to agglomerate particles in the soil limits its dispersibility and may cause soil slumping. Biochar (BC) with its porous structure and rich functional groups (Shaaban et al., 2018), can not only enhance the performance of nZVI, but also improve the soil texture and fertility. Su et al. [8,9] used BC-loaded nZVI (BC-nZVI) for the in situ remediation of Cr(VI)-contaminated soil. Compared to nZVI alone, BC-nZVI had better results for reducing the leachability and phytotoxicity of Cr(VI). Wan et al. [10] prepared a maize straw BC-loaded nZVI composite (MSBnZVI) with pH gradients that ranged from 4.0–8.0, and achieved a removal efficiency of 99%.

As nZVI and BC-nZVI have received increasing attention in soil remediation, the impact of application on the biodiversity of indigenous soil microorganisms should also be taken into account [11,12]. Indigenous microorganisms play an irreplaceable role in the biogeochemical cycling of the soil sphere [13]. For example, soil microorganisms play a key role in the transformation of Fe (Fe cycle) and the fate of Cr in the soil (Cr cycle). In addition, exogenous substances, such as nZVI and Cr alter the structure of the microbial community and, to some extent, influence the cycling of important elemental substances in the soil [14]. Biochar can generally increase soil bacterial abundance and improve their structure and communities in the soil. In contrast, the addition of nZVI inevitably affects soil ecology and may cause potential harm to indigenous microorganisms [15,16]. It has been shown that nZVI addition significantly alters the structure and composition of soil bacterial communities [17,18]. Anza et al. [19] documented that soil contaminants reduce microbial biomass and activity and that nZVI application negatively affects the microbial load of contaminated soil, but not uncontaminated soil. The authors concluded that microbial populations in contaminated soils were more sensitive to nZVI than those in uncontaminated soils. Recent studies have shown that BC can provide nutrients for soil microbial colonization and reduces the detrimental effects of nZVI on soil microorganisms [20]. However, relatively few reports have been published on the ecological effects of BC-nZVI on soil microorganisms during the remediation of Cr-contaminated soils. Therefore, it is important to study the remediation effect of BC-nZVI composites and the impact on the microbial ecology of Cr-contaminated soils to contribute to a comprehensive assessment of soil safety and ecosystem restoration functions.

In view of the above facts, the main objectives of this study were: (i) To prepare ramie biochar (BC), nZVI, and BC-nZVI to investigate the effects of different materials with different dosage and remediation times on the removal efficiency of Cr(VI) from soil, to explore the remediation factors, and to study the mechanism of morphological transformation of Cr in soil; (ii) to examine the effects of the reaction materials on soil texture, mainly by measuring soil pH and dissolved organic carbon; (iii) to investigate the mechanism of the effect of materials on microbial effects, to analyze the diversity and abundance of indigenous microorganisms, and to clarify the effect of synthetic materials on changes in the structure of indigenous microbial communities.

## 2. Materials and Methods

### 2.1. Chemicals and Materials

The chemicals were of analytical grade or higher and purchased from the Shanghai Guoyao reagent group, Shanghai, China. Ultrapure water (resistivity ≥ 18.25 MΩ·cm) was used in all the experiments. The preparation and characterization of ramie biochar (RBC600, biochar prepared at a pyrolysis temperature of 600 °C), nano-zero-valent iron (nZVI), and nano-zero-valent iron supported by ramie biochar (BC-nZVI) have been explained in our previous study [21].

### 2.2. Soil Used in the Experiment

Soil samples (0–20 cm) were collected from a field (E 30°6′59.017″, N 114°10′16.403″) located in Jiangxia District, Wuhan City, Hubei Province, China. The soil in this area has been cultivated for a long period of time and can be regarded as the typical representative of farmland. After manual removal of visible plant residues and roots, the soil samples were air-dried and ground to pass through a 2-mm sieve.

According to China’s soil environmental quality for agricultural land standard (GB 15618–2018), the Cr threshold level in soil is considered toxic with 350 mg/kg. The soil used in the present study was not Cr-contaminated, and therefore, we artificially spiked the soil with Cr to simulate Cr-contaminated soil. To prepare the Cr-spiked soil, 1 kg of pristine soil was added into 1 L solution of K_2_Cr_2_O_7_ (500 mg/L) and vigorously homogenized with ultrasonic treatment and continuous stirring for 30 min. Thereafter, the soil was air-dried and stabilized for 3 months prior to use in the experiment. The concentration of Cr in spiked soil before use in experiments was: Total Cr = 406.4 mg/kg; Cr(VI) = 76.25 mg/kg.

### 2.3. Soil Incubation Experiment

Three different remediation materials (RBC600, nZVI, and BC-nZVI) and five different dosing rates (1, 3, 5, 7, and 10 g/kg) were tested for the removal of Cr(VI). A total of eight treatments were designed for this experiment: (i) Soil without any additive (CK), (ii) RBC600, 10 g/kg (T1), (iii) nZVI, 10 g/kg (T2), (iv) BC-nZVI, 1 g/kg (T3), (v) BC-nZVI, 3 g/kg (T4), (vi) BC-nZVI, 5 g/kg (T5), (vii) BC-nZVI, 7 g/kg (T6), and (viii) BC-nZVI, 10 g/kg (T7). Three replicates were set up for each treatment and 50 g of air-dried soil and the corresponding material were packed into 300 mL plastic beakers and mixed well. The soil moisture content was maintained at 60% during incubation. The tops of the beakers were covered with porous plastic wrap to ensure ventilation and to minimize water evaporation. All of the beakers were placed in an electric incubator (ZXSD-B1430, Shanghai, China) at 25 ± 0.5 °C in the dark. The soil was sampled for analysis on days 1, 7, and 15.

### 2.4. Analysis of Soil Cr

Soil samples collected on days 1, 7, and 15 were air-dried, crushed, and passed through a 0.15 mm sieve in the laboratory. Determination of Cr(VI) was carried out according to a method described by Tan et al. [22]. The species of Cr in the soil were evaluated by sequential Tessier extraction methods [23]. All of the above extracts were filtered through a 0.45-mm membrane and analyzed using the Flame atomic absorption spectrophotometer (AAS700, PerkinElmer, Waltham, MA, USA).

### 2.5. Soil Physicochemical Analysis

The soil pH was determined using a pH meter (PB-10, Beijing, China) at a soil:ultrapure water ratio of 1:2.5 (*w*/*v*) [24]. The DOC of the soil was analyzed using a Vario TOC/TN Analyzer (Elementar, Hanau, Germany) as described by Shaaban et al. [25]. The cation exchange capacity of the soil was measured using an UV Spectrophotometer (UV-8000, Shanghai, China) by the hexamine cobalt trichloride spectrophotometric method [26]. Bioavailable Cr and Fe were determined by the DTPA extraction method.

### 2.6. Soil Microbial Analysis

The soil samples were sent to Shanghai Paisano Biotechnology Co., Ltd. (Shanghai, China). for genomic DNA extraction, purification, and sequencing. The partial sequences of 16S rRNA genes, including V3-V4 high variant region genes were amplified using universal primer (5′ACTCCTACGGGAGGCAGCA-3′) and reverse primer (5′GGACTACHVGGGTWTCTAAT-3′) amplification conditions: 98 °C, 2 min (initial denaturation); 98 °C, 15 s; 55 °C, 30 s; 72 °C, 30 s (for 25–27 cycles); 72 °C, 5 min (final extension).

### 2.7. Statistical Analysis

One-way ANOVA was used to analyze the data for the significance of differences. The sequences obtained from gene sequencing were assigned to each operational taxonomic unit (OTU) by 97% similarity, and the species abundance and diversity of the soil bacterial community were analyzed by dilution curves, Shannon, Coverage, Chao1, and other indices. Wayne diagrams, relative abundance analysis of dominant species, and principal coordinate analysis were used to reveal the changing patterns of bacterial community composition and structure. Bio-Rad CFX Manager software was used for statistical analysis.

## 3. Results

### 3.1. Remediation Effect of Cr(VI) in Soil

**Figure 1:**
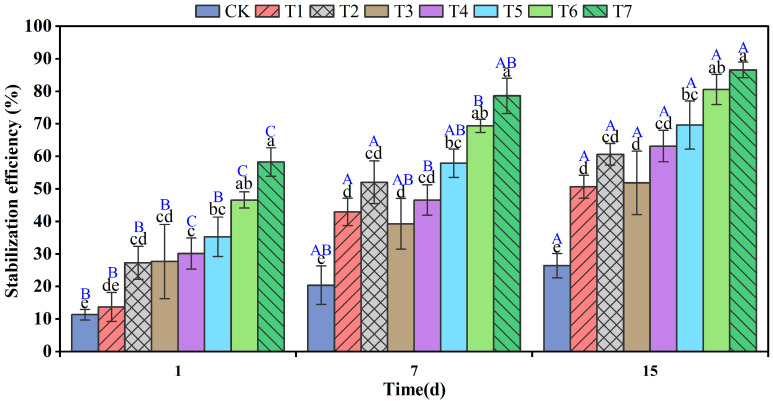
Effect of different times and treatments on the remediation of hexavalent chromium in soil (different capital letters represent significant differences between different times of the same treatment, different lowercase letters represent significant differences between different treatments at the same time, *p* < 0.05). (Soil without any additive (CK), RBC600, 10 g/kg (T1), nZVI, 10 g/kg (T2), BC-nZVI, 1 g/kg (T3), BC-nZVI, 3 g/kg (T4), BC-nZVI, 5 g/kg (T5), BC-nZVI, 7 g/kg (T6), BC-nZVI, 10 g/kg (T7)).

The effect of the eight treatments on the remediation of Cr(VI) in soil at different times is shown in Figure 1. One day after remediation, the stabilization of Cr(VI) in the soil of treatments CK and T1–T7 was 11.32%, 13.73%, 27.31%, 27.68%, 30.13%, 35.24%, 46.58%, and 58.25%, respectively. After 7 days of restoration, the stabilization of Cr(VI) in the soil of treatments CK and T1–T7 was 20.41%, 42.95%, 52.03%, 39.26%, 46.58%, 57.89%, 69.35%, and 78.60%, respectively. After the 15th day of soil sampling, the immobilized Cr(VI) in the soil increased to 26.39%, 50.64%, 60.63%, 51.81%, 63.16%, 69.61%, 80.55%, and 86.55% for the eight treatments, i.e., CK and T1–T7, respectively.

These results showed that a remediation time of 15 days was sufficient to achieve good remediation results, and the best remediation effect was achieved at an application rate of 10 g/kg.

### 3.2. The Speciation of Cr in the Soil

Studies have shown that the risk of heavy metal contamination in soils is not only related to the total amount of heavy metals, but also closely linked to their fugitive form. The form of deposition directly influences the transport and transformation processes of heavy metals [27], which in turn determines their biological effectiveness. The environmental activity and effectiveness vary considerably between the different forms present [28]. Among them, the exchangeable state is easily transportable and bioavailable; the stability of the carbonate bound state is related to soil pH, and a decrease in pH may lead to the release of heavy metals; the organic bound and Fe-Mn oxidized states are more stable than the first two and are mainly influenced by pH and Eh; the residue state is the most stable and is not easily released or bioavailable [29]. The environmental activity of soil heavy metal fugitive forms is as follows: Exchangeable > carbonate bound > Fe-Mn oxidized > organic bound > residue [30], and vice versa for environmental risk.

**Figure 2:**
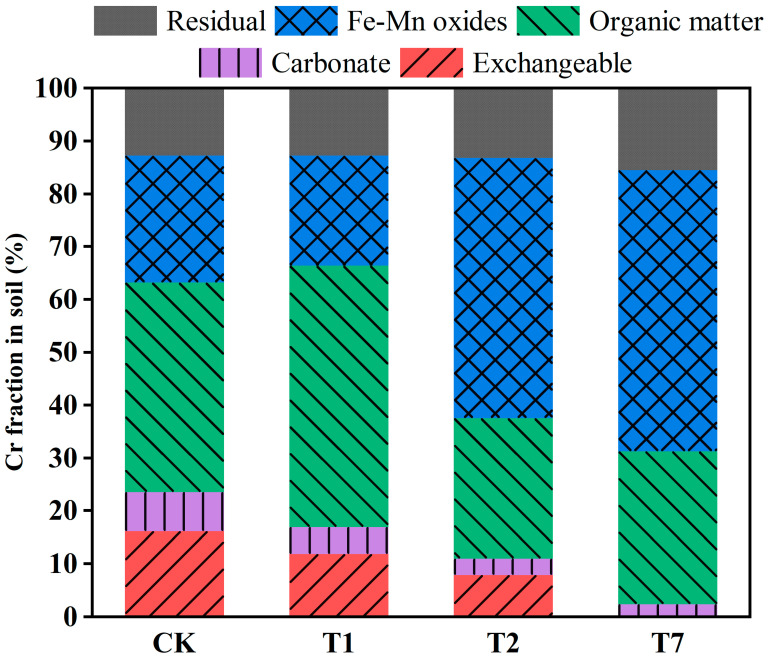
Effect of compound type on the distribution of chromium species in soil. (Soil without any additive (CK), RBC600, 10 g/kg (T1), nZVI, 10 g/kg (T2), BC-nZVI, 10 g/kg (T7)).

To further understand the remediation mechanism, we used a sequential extraction procedure (SEP) to examine the morphological changes in Cr, a heavy metal, in the soil after CK, RBC600, nZVI, and BC-nZVI treatments. Although the true morphology of Cr in the soil cannot be accurately characterized in the ratio between the various forms of Cr under different treatments, curing is beneficial to the analysis of the interaction between remediator materials and heavy metals and can be analyzed. As can be seen in Figure 2, Cr in CK was mainly present in the exchangeable (16.16%), carbonate bound (7.35%), organic bound (39.67%), Fe-Mn oxidized (24.05%), and residue (12.77%) states. The RBC600-treated soil, i.e., T1, had 11.85% exchangeable, 5.07% carbonate bound, 49.45% organic bound, 20.86% Fe-Mn oxidized, and 12.77% residue states. Compared to the change in Cr morphology in CK, the exchangeable state in T1 decreased by 4.31% and the organic bound state increased by 9.78%. This is probably due to the fact that the surface of RBC600 is rich in oxygen-containing functional groups and complexes with Cr(III) to produce the organic bound state, which is one of the important mechanisms by which BC can remediate Cr contamination in soil.

The nZVI-remediated soil, T2, had 7.88% exchangeable, 3.18% carbonate bound, 26.54% organic bound, 49.27% Fe-Mn oxidized, and 13.13% residue states. Compared to CK, the exchangeable state in T2 decreased by 8.28% and the Fe-Mn oxidized state increased by 28.41%. This is mainly due to the adsorption of Cr ions from the soil on the surface of the nZVI particles after the nZVI remediation, which complexes and precipitates with the surface Fe oxides as well as Fe hydroxides to form trivalent Cr-Fe hydroxides [31]. After the BC-nZVI remediation, the exchangeable state disappeared completely from the soil, i.e., T7, with 2.38% carbonate bound, 28.91% organic bound, 53.19% Fe-Mn oxidized, and 15.52% residue states. It can be found that almost all of the exchangeable state in T7 was transformed into Fe-Mn oxidized and residue states, in which the Fe-Mn oxidized state increased by 29.14% and the residue state increased by 2.75% compared to CK, which is mainly attributed to the synergistic effect of RBC600 and nZVI, and also indicates that BC-nZVI can immobilize the Cr in the soil more effectively.

### 3.3. Effect of BC-nZVI on Cr-Contaminated Soil Microorganisms

#### 3.3.1. Soil Bacterial Community Diversity Analysis

There are many microorganisms present in soils and they play a key role in maintaining the ecological balance of the system. At the same time, the sensitivity of soil microbial communities to external factors or anthropogenic disturbances can be used as a good indicator of soil ecological risk and health [32]. Research on soil microbial community diversity, changes in community structure, and succession patterns during soil remediation can reveal the mechanism of action of BC-nZVI reduction in the remediation of Cr(VI)-contaminated soil from a microscopic perspective.

**Figure 3:**
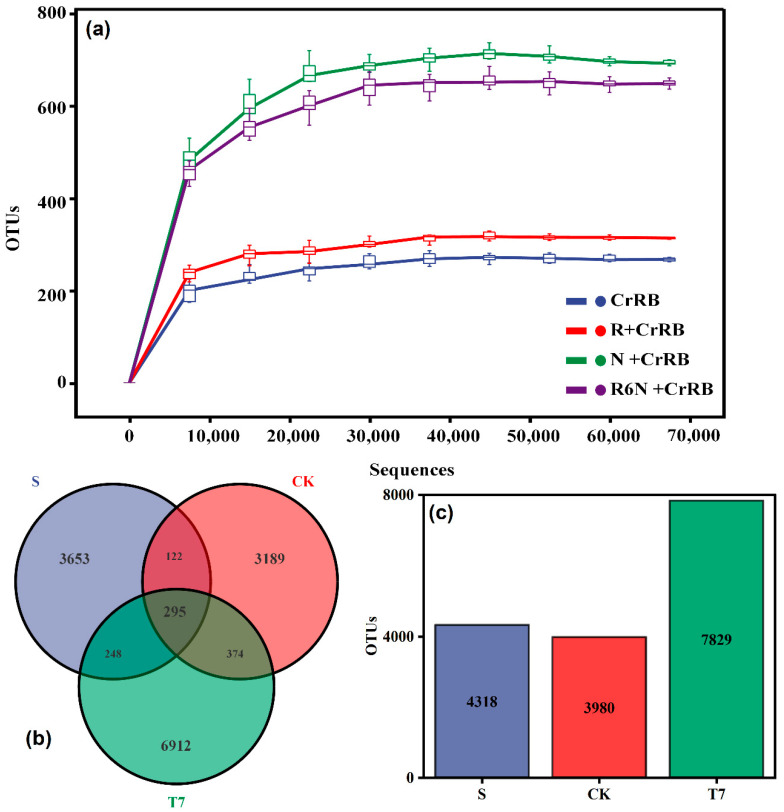
Soil sample bacterial community dilution curve (**a**); Venn diagram (**b**) and number of OTUs (**c**) of bacterial communities in different treatment fractions. (Unactivated original contaminated soil (S), soil without any additive (CK), BC-nZVI, 10 g/kg (T7)).

A total of 920,494 valid sequences and 519,286 high quality sequences were obtained by high-throughput sequencing of a total of eight soil samples from three groups, S, CK, and T7, which could be classified into 3980–7829 OTUs at 97% similarity level. As shown in Figure 3a, the dilution curves of all three groups of samples tended to be flat, indicating that the amount of data were reasonable and reliable, the sequencing depth of the samples met the requirements, and could complete the response of soil microbial community [33]. Moreover, it can be seen that the T7 group had the largest number of OTUs and the highest species richness, which can indicate that BC-nZVI had no toxic side effects and even promoted the metabolism of indigenous microorganisms to some extent.

The Venn diagram enables the number of co-occurring and individual species to be counted between groups of samples, thus providing a more visual representation of the composition and similarity of OTUs as well as an overlap of the flora under different conditions, which reflects the distribution and changes in community structure under different stresses [34]. In Figure 3b,c, the total number of OTUs for the three groups of samples, S, CK, and T7, were 4318, 3980, and 7829, respectively. Of these, 295 OTUs were detected jointly, indicating that some microorganisms were always present in different treatments; the number of OTUs unique to each group of samples was 3653, 3189, and 6912, respectively, which indicates that different treatments produced their appropriate unique strains. The T7 treatment showed higher OTUs compared to S and CK, indicating that BC-nZVI contributes to the development of soil biodiversity, improvement of soil structure, and maintenance of soil fertility.

Heavy metals are important factors affecting microbial communities in the soil environment [35,36], and these effects are usually reflected in the abundance and diversity of microbial communities [37]. The abundance and diversity of microbial communities can be reflected by the α-diversity index, which includes abundance, diversity, and homogeneity, e.g., Chao1 [38], Shannon [39], Simpson [40], and Pielou [41], etc. The α-diversity indicators for each group of samples are shown in Table S1. From the data in the table, it can be seen that the Chao1 and Observed species indices of the samples in T7 treatment were significantly higher than those of S and CK (*p* < 0.05), and the greater the Chao1 and Observed species indices, the higher the richness of the community. Therefore, the T7 community contained the greatest number of OTUs and the greatest richness of the community. For the Simpson index value, the larger the Simpson index value, the lower the community diversity, and the larger the Shannon value, the higher the community diversity, which indicates that T7 has the highest community diversity. The higher the Pielou evenness index, the more homogeneous the community, thereby the T7 treatment has the most homogeneous community.

#### 3.3.2. Soil Bacterial Community Structure Analysis

Soil microorganisms can alter the composition and structure of the corresponding microbial communities, and the analysis of soil microbial community composition can assess the response mechanisms associated with Cr fixation via BC-nZVI and provide a more detailed picture of its possible impact on the soil microbial community.

The abundance of microbial communities in soil samples at the phylum level varied as shown in Figure S2a, with Firmicutes, Actinobacteria, and Proteobacteria as the dominant phylum (relative abundance > 1%). In the S and T7 treatments, Firmicutes was the most dominant group with relative abundances of 89.33% and 90.37%, respectively. In contrast, in the CK treatment, the first dominant phylum was Actinobacteria, with a relative abundance of 49.49%, and the next dominant phylum consisted of Firmicutes (27.81%) and Proteobacteria (21.64%), respectively. Therefore, the composition of the bacterial communities of the three groups was essentially similar at the phylum level, but the abundance distribution had some variability, especially in the CK group. This further confirms that BC-nZVI did not have a detrimental effect on soil microbiology. Figure S2b shows the changes in the relative abundance of soil microorganisms at the phylum level, combined with the changes in taxonomy at the phylum level to further reveal the effect of BC-nZVI on microbial composition. It can be seen that Bacilli, Actinobacteria, and Clostridia are the dominant phyla (relative abundance > 1%) in the three groups, all of which are reported to have Cr(VI)-reducing functions. It was found that the community structure and abundance distribution at the phylum level were very similar to those at the phylum level, with Bacilli as the most dominant group in the S and T7 groups, with relative abundances of 87.14% and 82.08%, respectively. In the CK group, the first dominant group was Actinobacteria with a relative abundance of 49.23%, while the second dominant group consisted of Bacilli (25.20%) and Alphaproteobacteria (25.20%). It can be seen that the proportion of Clostridia (7.73%) in T7 increased compared to both S (2.09%) and CK (2.52%), which is mainly caused by the addition of BC-nZVI. In addition, earlier studies reported the identification of Clostridia as heterotrophic Fe(III)-reducing bacteria [42]. Therefore, the increase in Clostridium may favor the elimination of the nZVI passivation layer, and thus allow for a better reaction with Cr contaminants.

**Figure 4:**
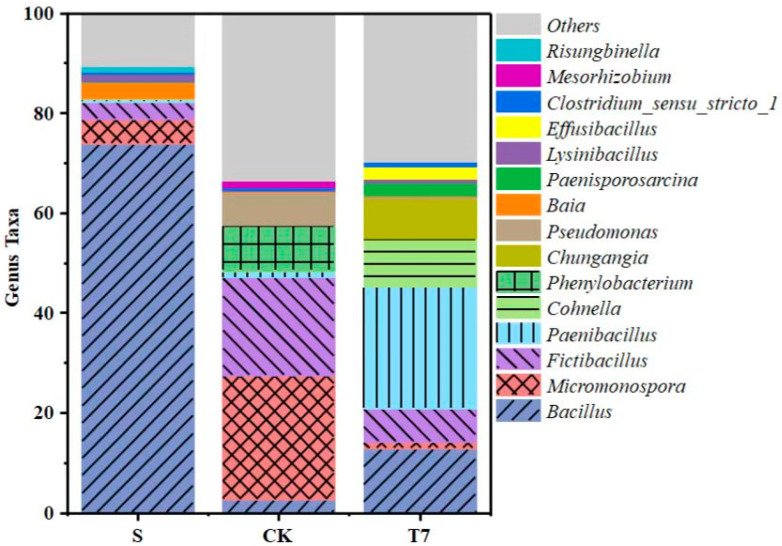
Relative abundance of bacterial communities in soil samples at the genus level. (Unactivated original contaminated soil (S), soil without any additive (CK), BC-nZVI, 10 g/kg (T7)).

To reveal more deeply the structural variability of the microbial communities in the different groups of samples, we analyzed them at the taxonomic level of the genus, and the variation at the genus level was more evident in the samples of each treatment group, with the composition of the dominant communities (relative abundance > 1%) shown in Figure 4. The dominant genera in the Group S samples were Bacillus (73.86%), Micromonospora (4.82%), Fictibacillus (3.52%), Baia (3.39%), Lysinibacillus (1.50%), and Risungbinella (1.11%), most of which are both Cr(VI)-tolerant and Cr(VI)-reducing bacteria. In the CK group compared to the S group, Bacillus decreased sharply to 2.65% and Micromonospora (24.81%) increased as the first dominant genus and typical metal-reducing bacterial genus [43]; Fictibacillus (19.57%) was the second dominant genus and it was reported that (Fictibacillus, 19.57%) was the next dominant genus, which was reported to be able to resist and reduce Cr(VI) [44]. In addition, four new dominant genera, Phenylobacterium (8.98%), Pseudomonas (6.85%), Paenibacillus (1.28%), and Mesorhizobium (1.45%) were added. In the T7 treatment, Paenibacillus (24.23%) was overwhelmingly dominant in the samples. Of note, the T7 group was not only highest in abundance and diversity of microbial communities, but also the presence of dominant groups was not found in other samples, such as Hungangia (7.85%), Effusibacillus (2.44%), Cohnella (9.77%), and Paenisporosarcina (2.60%), indicating that BC-nZVI reduced the environmental stress caused by Cr pollution and restored soil microbial diversity.

**Figure 5:**
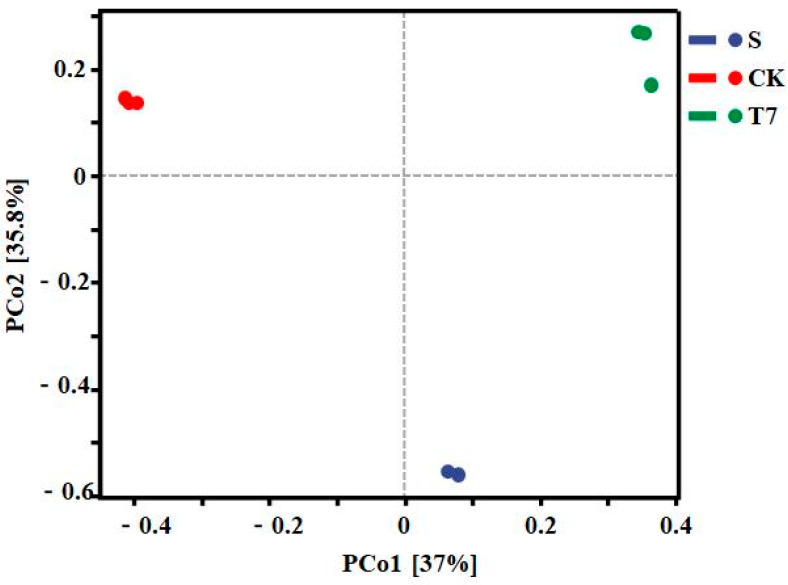
PCoA plots of bacterial communities in soil samples with different treatments. (Unactivated original contaminated soil (S), soil without any additive (CK), BC-nZVI, 10 g/kg (T7)).

Principal coordinate analysis (PCoA analysis) is one of the most classical methods of unconstrained ranking analysis [45]. PCoA takes sample distances as a whole into account and can be used to study similarities or differences in the community composition of soil sample species. Therefore, to further understand the differences in bacterial community structure under different treatments, principal coordinates were analyzed at the OTUs level for each group of samples. As shown in Figure 5, the variance explained by the first principal component (PCoA1) and the second principal component (PCoA2) were 37% and 35.8%, respectively. Each point in the graph represents a sample, the different colors represent the grouping of the samples and the distance between the points represents the degree of variation in the microbial communities of the samples. Here, the greater the distance, the greater the variation in the microbial communities between the samples. Therefore, it can be seen that the soil samples from different groups are clearly separated and far apart from each other, indicating that the different treatments made the bacterial communities in the soil very different and that the structure of the bacterial communities changed significantly.

**Figure 6:**
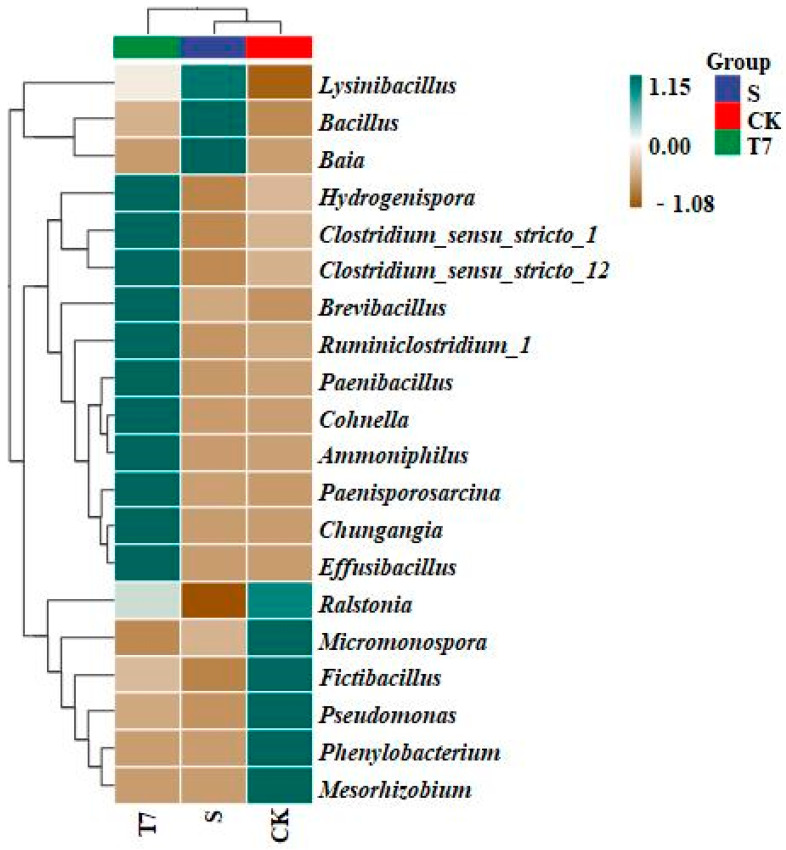
Heat map analysis of clusters at the genus level of soil samples. (Unactivated original contaminated soil (S), soil without any additive (CK), BC-nZVI, 10 g/kg (T7)).

To further compare species composition differences between samples and to achieve a demonstration of trends in species abundance distribution across samples, species composition analysis was carried out using a species abundance clustering heatmap. As shown in Figure 6, the cladistic analysis of the heat map visualizes the phylogenetic relationship of OTUs between the samples, and the heat map shows the clustering tree for the different treatments, with the distances of the branches and the distances of the clusters in the cluster analysis representing the variability in the bacterial structure of the samples. It can be seen that there are significant differences in microbial structure between the S, CK, and T7 treatments, which is consistent with the results of relative abundance and with the PCoA conclusions.

### 3.4. Effects of BC-nZVI on Soil Chemical Properties

**Figure 7:**
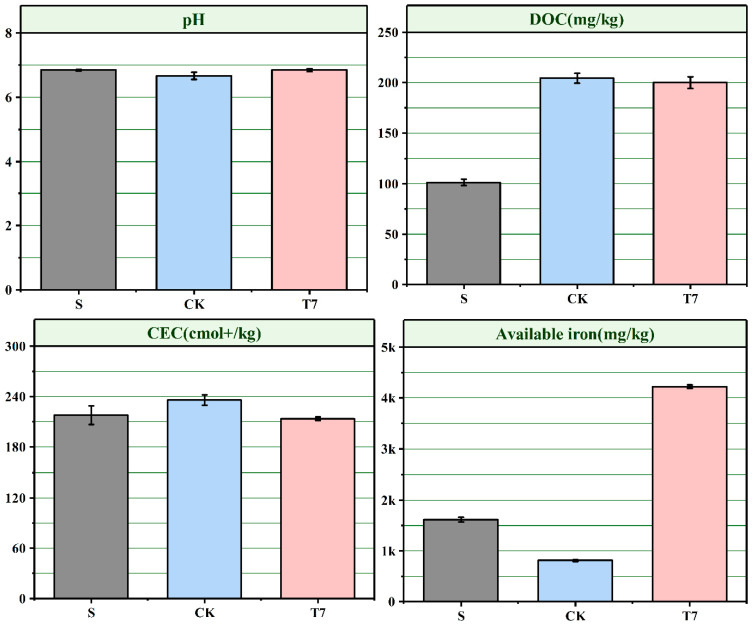
Effect of BC-nZVI on soil properties. (Unactivated original contaminated soil (S), soil without any additive (CK), BC-nZVI, 10 g/kg (T7)).

Soil is a complex non-homogeneous mixture [13], and the mobility of Cr in soil is closely related to soil texture, soil microorganisms, and other environmental factors [46]. To investigate the changes in soil properties before and after BC-nZVI remediation, the pH, DOC, CEC, and effective state Fe and effective state Cr of the original contaminated soil (S), the control treatment (CK), and the Cr-contaminated soil (T7) after BC-nZVI remediation were measured in this experiment and are shown in Figure 7 and Figure S1, respectively.

Soil pH is an important factor affecting the biological effectiveness of heavy metals in soil [26]. After the treatment, the pH of the three soils, S, CK, and T7, was 6.84, 6.66, and 6.84, respectively. The increase in soil pH of 0.18 in T7 compared to the control treatment may be due to the reduction reaction of iron decay with Cr(VI), during which H^+^ was consumed, resulting in an increase in OH- [47]. Moreover, it may be due to the release of alkaline salts to the soil by RBC600 resulting from the release of alkaline salts to the soil by RBC600 [48]. Soil organic carbon is one of the indicators of soil fertility and can complex soil heavy metals, and its active component DOC is an important indicator [49]. The DOC contents of the three soils, S, CK, and T7, were 101.2, 204.5, and 200.1 mg/kg, respectively. The soil DOC contents of both CK and T7 were twice as high as those of S. The increased DOC in CK may be due to the proliferative, locally intensive activity of soil microorganisms. The increase in DOC in T7 may be due to the increased aromatization and humification of soil after the addition of RBC in BC-nZVI to the soil, resulting in an increase in soil DOC content. The CEC represents the sorption capacity of soil colloids for various cations and is a non-negligible parameter affecting the stability of heavy metals in soil. The CEC of the three soils, S, CK, and T7, was 218.05, 236.03, and 213.66 cmol^+^/kg, respectively. Compared to the control treatment, the soil CEC value of T7 was reduced by 22.37, probably due to the low activity of nZVI after the reaction which allowed the release of Fe^2+^ to be limited, thus exhibiting a lower cation exchange. Fe is one of the major elements in soil and the increase in effective Fe content has a positive effect on enhancing soil microorganisms [12] with 1608.27, 806.51, and 4224.49 mg/kg of effective Fe found in the three soils S, CK, and T7, respectively. Therefore, T7 exhibited a higher Fe content, which was mainly due to the release of excess Fe from nZVI during the remediation process. This was mainly due to the release of excess Fe from nZVI during the remediation process, resulting in an increase in Fe content in the soil.

Specifically, changes in soil pH, DOC, CEC, and effective iron content following the application of BC-nZVI suggest that BC-nZVI can influence the ability of heavy metals to bind to the soil by altering the physicochemical properties of the soil to reduce the effective state of chromium in the soil (Figure S1).

## 4. Discussion

Soil microbial richness and diversity are considered to play a critical role in maintaining stability in ecosystem productivity and function and in mitigating stresses and disturbances [50], and changes in them may have important implications for soils [51]. Previous studies have used electrokinetic techniques, phytoremediation [52], microbial remediation, and microbial dye batteries [53] to remediate chromium-contaminated soils, and many of these methods have been undiscovered. In the present study, which enriched chromium reducing bacteria in soil and indigenous microbial diversity counted by high-throughput gene sequencing, we obtained the α-diversity index of bacterial communities in chromium-contaminated soils (Table S1). Our approach identified conventional taxa that were present in enrichment cultures (Figure 4), and revealed some rare CrRB community taxa (e.g., Hungangia, Effusibacillus, Cohnella, Paenisporosarcina, etc.). In addition, many “Unclassified” sequences were identified in this study, suggesting that these paddy soils contain a quantity of unknown CrRB.

According to previous studies, Firmicutes, Actinobacteria, and Proteobacteria are typically the dominant treatments among the CrRB communities in Cr-contaminated soils [44,54,55,56]. Previously, it has been studied that Proteobacteria and Firmicutes were also the dominant phyla in the gut, gonad, and hepatopancreas of female shrimp, while Firmicutes, Actinobacteria, and Proteobacteria were also found in Colombian Caribbean deep-sea sediments and found in the soil of the Lake Elton area. Meanwhile, members of other phyla, such as Acidobacteria, Ascomycota and Basidiomycota, Chytridiomycota, Chlorophyte, Omoarchaea, and Broadarchaea have been described in different chromium-contaminated soils, and they may play a key role in C and N cycling. However, these taxa are rarely classified as CrRBs, thus other unknown CrRB phyla may be the subject of future research.

In the present study, Firmicutes, Actinobacteria, and Proteobacteria were dominant in the soil, and there were certain differences in the relative abundance of these groups. In the S and T7 groups, bacterial cell walls of Firmicutes contain a peptidoglycan layer and are able to form budding spores under stressful conditions (e.g., lack of nutrients or presence of contaminants), protecting the cells from environmental damage [57]. In the CK group, the first dominant flora was Actinobacteria, and the second dominant flora were Firmicutes and Proteobacteria. These results confirmed that BC-nZVI had no adverse effect on soil microecology.

In addition, the increase in community richness and diversity in the T7 group was mainly attributed to the incorporation of RBC600-nZVI, which reduces the invasive effects of Cr [34], while changes in soil environmental conditions stimulated microbial growth and activity [58,59,60]. Moreover, RBC600-nZVI provides some nutrients and survival habitat for microorganisms [61,62,63].

It has been previously reported that the diversity of bacterial community is greatly influenced by soil properties. In the present study, there were significant differences in the available iron content in CK and T7 soils, and significant differences in the DOC content in S and T7 soils, suggesting that the diversity of CrRB in chromium-contaminated soils was affected by soil properties. The increase in DOC in T7 may be due to the increase in soil aromatization and humification after RBC in BC-nZVI was added to the soil, resulting in the increase in soil DOC content.

The significant difference (*p* < 0.05) between the restoration effect of CK on day 15 compared to that on day 1 could be explained by the enrichment growth of indigenous microorganisms during soil cultivation, where some of the Cr(VI) was reduced to Cr(III) by the action of microorganisms with the Cr(VI) reducing ability or possibly by the reduction in some organic matter that is still present in the soil itself [3,11,64]. Moreover, the remaining treatments reached maximum Cr(VI) stabilization rates on day 15.

The stabilization of Cr(VI) by BC-nZVI (T7) was significantly higher (*p* < 0.05) than RBC600 (T1) and nZVI (T2) at the same dosing rate. The possible reasons for this are that BC reduces Cr(VI) through reactive treatments on its surface, disordered PAHs provide π-electrons for Cr(VI) reduction, and compounds, such as -OH in phenols and alcohols are oxidized as proton donors. In addition, the resulting C=O or -COOH can provide binding sites for Cr(III) as a means to stabilize Cr(VI). The nZVI itself is magnetic and tends to adsorb and agglomerate into large particles, reducing its own specific surface area. The large particles of nZVI tend to react and passivate with substances in the environment due to the more reactive surface layer, leading to unsatisfactory remediation results.

Moreover, the solid form of nZVI itself is difficult to migrate in soil pores and is less mobile, making it difficult to reach the Cr(VI) adsorbed by the soil. The increased stabilization of Cr(VI) by BC-nZVI may be due to the synergistic effect of RBC600 and nZVI [4,17]. The presence of RBC600 improves the dispersion and stability, increasing the specific surface area, and thus providing more sorption sites [18]. Furthermore, RBC600 enhanced the mobility of BC-nZVI in the soil, thus allowing for the reduction in more Cr(VI). The effect of BC-nZVI (T3–T7) on the remediation of Cr in the soil increased with the increasing dosage, which was due to the fact that the higher dose of BC-nZVI provided more active sites [19].

## 5. Conclusions

This study showed that BC-nZVI at 10 g/kg was most effective at reducing 86.55% of the Cr(VI) in the soil after 15 days of application, which is significantly higher than the application of RBC600 (50.64%) and nZVI (60.63%). The exchangeable state in the restored soil was almost entirely converted into the Fe-Mn oxidized and residue states. High throughput sequencing results showed that BC-nZVI had no negative impact on soil microorganisms and can even increase the abundance and diversity of indigenous bacteria by reducing toxic effects of Cr, changing soil properties, and providing habitat for survival.

## Data Availability

The data presented in this study are available on request from the corresponding author.

## Supplementary Materials

The following supporting information can be downloaded at: https://www.mdpi.com/article/10.3390/nano12193541/s1, Figure S1: Effect of RBC600-nZVI on the effective state of chromium in soil. (Unactivated original contaminated soil (S), Soil without any additive (CK), RBC600-nZVI 10 g/kg (T7); Figure S2: Relative abundance of bacterial communities in soil samples at the phylum (a) and phylum (b) levels. (Unactivated original contaminated soil (S), Soil without any additive (CK), RBC600-nZVI 10 g/kg (T7); Table S1: α-diversity index of bacterial communities in chromium-contaminated soils.
