# Supplementary material for: Remediation of Cr(VI)-Contaminated Soil by Biochar-Supported Nanoscale Zero-Valent Iron and the Consequences for Indigenous Microbial Communities
Source: Nanomaterials (Basel). 2022 Oct 10;12(19):3541. doi: 10.3390/nano12193541 (PMC9565499; doi:10.3390/nano12193541)
Supplement: Supplementary file 1 [file nanomaterials-12-03541-s001.zip › nanomaterials-1946639-supplementary-revised.pdf]

nanomaterials

*Supplementary Materials*

# Remediation of Cr(VI)-Contaminated Soil by Biochar-Supported Nanoscale Zero-Valent Iron and the Consequences for Indigenous Microbial Communities

**Jianwei Yang [1], Xiangpeng Tan [1], Muhammad Shaaban [2], Yajun Cai [1], Buyun Wang [1] and Qi'an Peng [1,*]**

[1] School of Environmental Engineering, Wuhan Textile University, Wuhan 430073, China
[2] Key Laboratory of Mountain Surface Processes and Ecological Regulation, Institute of Mountain Hazards and Environment, Chinese Academy of Sciences, Chengdu 610041, China
* Correspondence: pqa2002_@163.com

**Table S1.** $\alpha$-diversity index of bacterial communities in chromium-contaminated soils.

| Sample | Chao1 | Observed species | Pielou's evenness | Shannon | Simpson |
|---|---|---|---|---|---|
| S | 2673.08 ± 51.83$^{b}$ | 2612.55 ± 63.75$^{b}$ | 0.71$^{a}$ | 8.05 ± 0.08$^{a}$ | 0.97$^{ab}$ |
| CK | 1987.45 ± 174.38$^{c}$ | 1736.10 ± 145.01$^{c}$ | 0.58 ± 0.04$^{b}$ | 6.24 ± 0.54$^{b}$ | 0.93 ± 0.03$^{b}$ |
| T7 | 3266.92 ± 8.37$^{a}$ | 3198.04 ± 3.24$^{a}$ | 0.72 ± 0.01$^{a}$ | 8.35 ± 0.09$^{a}$ | 0.98$^{a}$ |

Note: Different letters in the table indicate significant differences in the samples ($p < 0.05$).

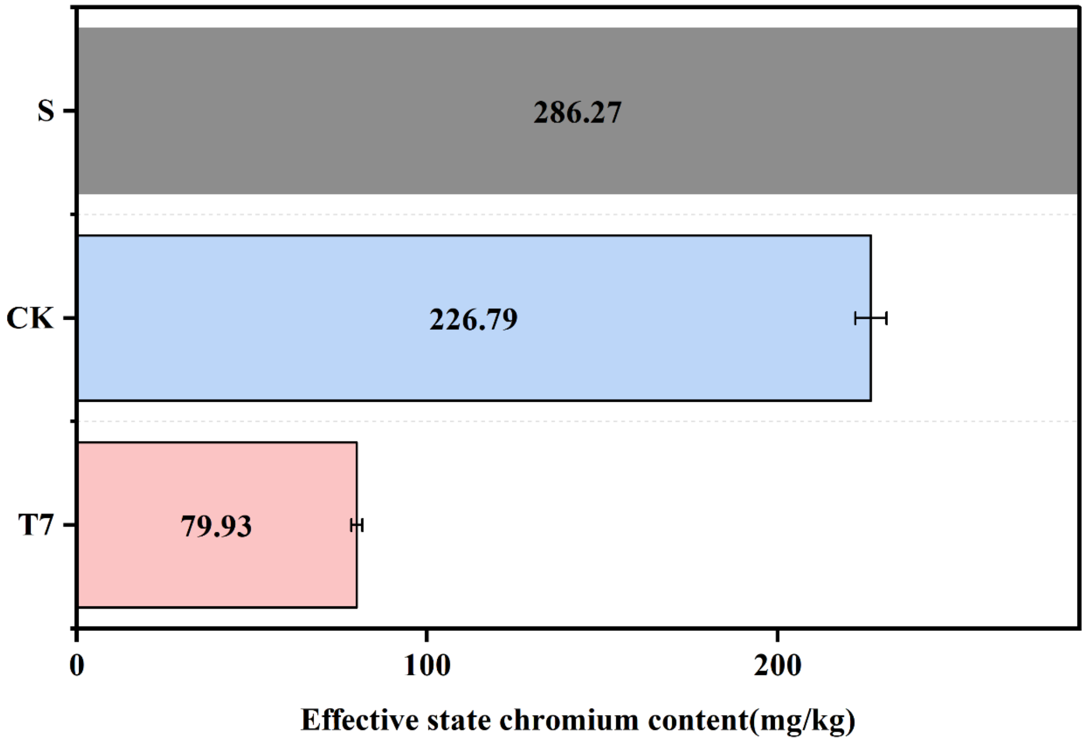


**Figure S1.** Effect of RBC600-nZVI on the effective state of chromium in soil. (Unactivated original contaminated soil (S), Soil without any additive (CK), RBC600-nZVI 10 g/kg (T7).

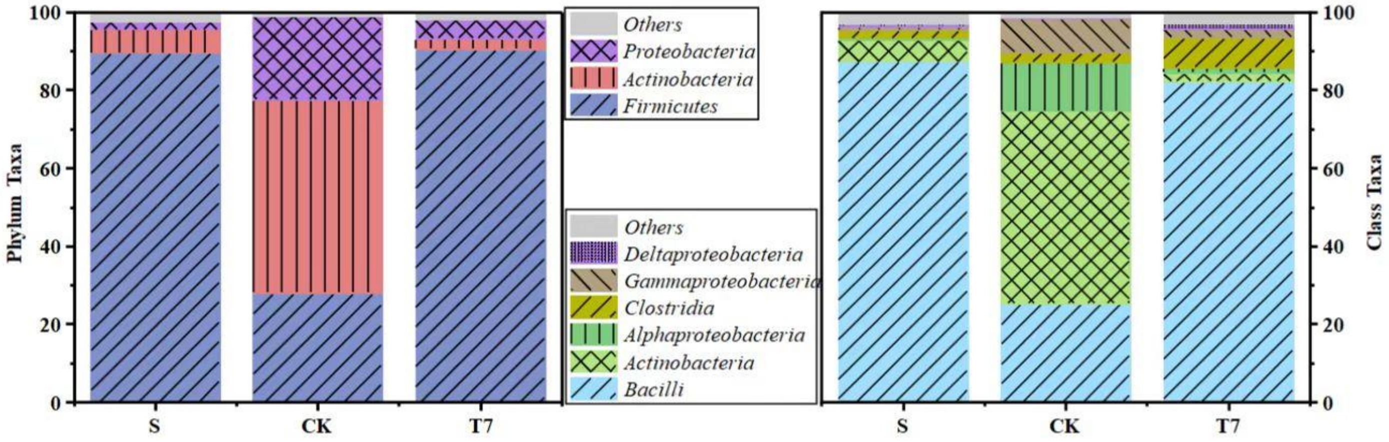


**Figure S2.** Relative abundance of bacterial communities in soil samples at the phylum (a) and phylum (b) levels. (Unactivated original contaminated soil (S), Soil without any additive (CK), RBC600-nZVI 10 g/kg (T7).
